# Motivations for adolescent offending and truancy from school: retrospective interviews with adults recently released from a custodial prison sentence in England

**DOI:** 10.1016/j.heliyon.2022.e09762

**Published:** 2022-06-20

**Authors:** Stephen Filkin, Dara Mojtahedi, Dominic Willmott

**Affiliations:** a University of Huddersfield, Huddersfield, UK; b Loughborough University, Leicestershire, UK

**Keywords:** Delinquency, Truancy, Educational disengagement, Youth offending, Criminal identity

## Abstract

This qualitative study considers the development of adolescent offending and examines a range of potential causes rooted in the issues of truancy, peer pressure, and educational and parental disengagement. Ten adult offenders recently released from prison were accessed through a probation service in the North West of England. Participants (*M* age = 35.2, *S.D* = 8.51) were interviewed about the indictable offences that they perpetrated between the ages of 12–16. Thematic analysis uncovered several key themes related to substance misuse and broader enjoyment of risk-taking behaviours, financial gain and the desire to develop a recognised criminal status, alongside fear and rejection of authority. In general, educational disengagement led to stronger associations with anti-social peers from whom acceptance was sought and offending identities were constructed around. Longer-term consequences of time spent with anti-social peers included substance abuse, more serious criminality, and increased risk-taking behaviour. The implications of the findings are discussed in the context of early interventions.

## 1 Introduction

A recent Ministry of Justice (2022) report examining crime rates in the United Kingdom (UK) displayed rates of reoffending were highest for offenders under that age of 18. The figures showed that 34.1% of juvenile offenders convicted for a criminal offence between January–March 2020 reoffended within 12 months of the original offence. According to the same report, reoffending juveniles perpetrated on average 3.6 offences during that period. With regard to the type of offence committed, adolescents who committed robberies (40.3%) and fraud (39.4%) were those most likely to reoffend. Similar gradual upward trends in juvenile delinquency, offending and recidivism rates are also observed elsewhere in the world, including throughout North America (Drury et al., 2020; Snyder, 2005), South East Asia (Shagufta et al., 2015; Sherretts and Willmott, 2016) and Eastern Europe (Debowska et al., 2015; Rode, 2014). Together, these figures highlight a growing issue faced by the criminal justice system in reducing juvenile delinquency and adolscent offending.

Whilst sociological factors undoubtedly account for children and young people’s early involvement in deviant and criminal behaviour, psychological research has identified a number of psychosocial predictors among such populations. Examples include deficits in social control and impulsivity (Beaver et al., 2015; Gallupe et al., 2015), low self-esteem (Spink et al., 2017), developmental disorders (Devine and Mojtahedi, 2021; Slaughter et al., 2019), trauma exposure (Willmott et al., 2018, Willmott et al., 2018; Wilson et al., 2013; Woodfield et al., 2019) and first hand experience of violence, abuse and neglect during childhood (Boduszek et al., 2019; Debowska et al., 2018, 2021). A range of more sociodemographic factors are also frequently associated with such offending outcomes during adolescent years including; gender (Day et al., 2014; Fagan and Lindsay, 2014; Johnson and Gilligan, 1983), parental and peer demographics (Evans et al., 2014; TenEyck and Barnes, 2015), socioeconomic status (Agnew et al., 2008); and a broad range of educational criteria including educational attainment, engagement, suspension from school and IQ (Boduszek et al., 2012; 2013; Mears and Cochran, 2013; Mojtahedi et al., 2017; Murray and Farrington, 2010). Likewise, peer relationships with deviant and criminally active individuals, as well as involvement or broader association with a criminal gang, are also found to be important determinants of wide-ranging juvenile crime and delinquency, across time and place (Boduszek et al., 2015; Mann et al., 2020; Shore, 2014; Spink and Woodfield, 2019; Willmott and Ioannou, 2017). Whilst this list is by no means exhaustive and it is widely understood that juvenile delinquency cannot be attributed to any single causal factor and is likely the outcome of a combination of a broad spectrum of sociological factors, researchers have consistently highlighted parental and educational neglect as important determinants of some adolscents decisions to engage in anti-social and criminal behaviours (Cohen, 1977; Dahl, 2015; Gottfredson and Hirschi, 1990; Hoeve et al., 2009; Ryan et al., 2013).

One feature (expressed or otherwise) often found to underpin adolescent delinquency is a lack of sustained investment in children at what is recognised as being the most impressionable time of their lives (Wright et al., 2001). This investment may begin with the parents, though must continue with committed multi-agency collaboration led in many instances by primary and secondary educational institutions. This approach seeks to provide a valid assessment of needs on a case-by-case basis to promote societal integration and the adoption of pro-social lifestyles and behaviour (Knight and Stout, 2009; Wong, 2013). Recognising the importance of educational institutions as a moderator of pro-social or anti-social lifestyles and behaviour, it is therefore necessary to consider the development of offending identities by examining issues of truancy, peer pressure, and general educational disengagement.

### 1.1 Truancy and deviance

Truancy can be operationally defined as ‘the habitual engagement in unexcused absences from school’ (Zhang et al., 2007, p. 245). The act of continuous absenteeism has been identified as a precursor to criminality (Lehr et al., 2004; Loeber and Farrington, 2000; McCluskey et al., 2004). Furthermore, disengagement with the educational process is associated with lower levels of achievement within the classroom (Havik et al., 2015), and various social, emotional and behavioural difficulties once widely referred to as ‘maladjustment’ (Carroll, 2013; Henry, 2007; Lim and Lee, 2016). Where a child’s interest is not engaged, the result is likely to be difficulties concentrating, non-participation and reduced attendance (Tennant, 2004; Vierhaus et al., 2016). As Havik et al. (2015) reported, effective classroom management is therefore key to enhancing pupil engagement. Effective teachers build environments where motivation is high and peer support prevents isolation and bullying—two strong predictors of truancy (Attwood and Croll, 2006; Gastic, 2008). Research suggests classroom disengagement is an important determinant of student wellbeing and self-concept as well as validation seeking through risk-taking behaviours (Curcio et al., 2016; Dishion and Tipsord, 2011; Reynolds and Crea, 2015; Richard et al., 2010). As Dahl (2015) highlights, decades of research from a variety of disciplines, link truancy to educational disruption and exclusion. This in turn increases the likelihood varied criminal behaviour and habitual substance misuse (Henry and Thornberry, 2010).

Likewise parental disengagement (sometimes termed lack of parental supervision) is also frequently related to juvenile truancy and delinquency (Breda, 2015; Collins et al., 2014; Hutchinson et al., 2009) and development of longer term of criminogenic thinking (Gonzalez et al., 2013; Hoeve et al., 2009). A child’s emotional stability and maturity develop according to the quality of domestic interactions and, if there are deficits within the home environment, there is likely to be a breakdown in social growth and educational receptivity (Thompson and Little, 1983). The dual impairments of parental and educational disengagement often result in a lack of supervision that in turn limits behavioural regulation and adherence to social and legal boundaries (Barnes and Farrell, 1992; Sogar, 2017; Unnever et al., 2004).

Another factor associated with truancy is substance abuse, Research suggests that truancy increases the likelihood of drug taking behaviour and more sustained substance misuse (Hallfors et al., 2002). Substance misuse for some young people leads to a rapid increase in the number of deviant behaviours and criminal offences that they engage in. Although there remains disagreement regarding the causal link between these factors, with some research suggesting that truancy is predictive of substance abuse (Bryant et al., 2003; Engberg and Morral, 2006); whilst others assert substance misuse leads to increased rates of truancy (Baumrind and Moselle, 1985; Godley, 2006). In many cases disrupted parental and educational engagement are found to most strongly predict truancy from school (Buu et al., 2009; Willoughby et al., 2004). Whatever the direction of the relationship between truancy from school, substance (mis)use and juvenile deviance and criminal behaviour, what is clear from much of the existing published literature, is that such factors are often present when retrospectively examining criminality within adulthood.

Another correlate of both truancy from school and delinquent behaviour is the presence of deviant peers (Asscher et al., 2013). Research frequently concludes that children, whom lack a strong attachment with the family unit and primary care givers, often look to peer groups for social acceptance. Studies show parental over-control or rejection can result in children experiencing heightened anxiety or perceived insecurity (Bradford et al., 2016; Brassard et al., 1987). The deficit in parental relations and subsequent involvement with deviant peer-groups are therefore consistent predictors of juvenile delinquency and offending. In fact, one dominant theory known as the Integrated Psychosocial model of Criminal Social Identity [IPM-CSI] (Boduszek et al., 2016) has found vast empirical evidence to support the proposition that offending behaviour and identities are typically underpinned by the individual’s bond and positive regard held towards their deviant and offending peers (Boduszek et al., 2013, 2016, 2021; Sherretts and Willmott, 2016; Spink et al., 2020).

Youth exposure to violence is another important predictor of truancy and criminal behaviour among adolescents (Dahl, 2015; Karcher, 2002; Peguero et al., 2016; Shulman et al., 2017). Violence exposure at home or outside of the domestic environment is often associated with subsequent problematic behaviour at school, the onset of criminality not encountered beforehand and adverse mental health outcomes (Baker et al., 2001; Bevan and Higgins, 2002; Cappell and Heiner, 1990; Dlamini et al., 2017; Farrington and Loeber, 2000; Sharratt et al., 2022).

There exists a vast literature examining correlates and casual factors underpinning truancy and offending behaviour among adolescent populations. However, despite many predominately quantitative studies examining the correlates of such outcomes from archival data or through cross-sectional self-report questionnaires, there remains a lack of research which has sought to retrospectively understand the aetiological basis of such behaviours through first-hand accounts with those who engaged in the behaviour during childhood and whom are now adults (Dahl, 2015). Existing research is also yet to directly examine pupil disengagement on an individual basis through in-depth qualitative interviews where less prominent, but nonetheless important, determinants of delinquent behaviour may be advanced by with those who involved in such behaviour during adolescence.

### 1.2 Current study

This study sought to consider the development of offending behaviour and identity by examining the issues of truancy, peer pressure, educational and parental disengagement through retrospective interviews with adults who had once been involved with juvenile offending. The study asked participants to account for their domestic, truanting and known offending histories. It was anticipated that the passage of time and process of maturation of the adult respondents would allow them to reflect on their upbringing with a greater understanding of the factors that they feel contributed to their decision to engage in truancy and criminal activity. The main aims of the present study were therefore to identify casual determinants of adolescent truancy and offending to assist in the early identification of risk factors, as perceived by adult offenders retrospective self-assessments.

## 2 Materials and methods

### 2.1 Participants

Access to a pool of candidates recently released from prison and attending probation in the city of Liverpool (UK), was facilitated through two probation case workers. A total of 25 prospective participants were invited to take part in the study at their probation facility, identified by the two probation case workers who they worked with post-release from custody. All 25 were given a participant information sheet and simply asked to return this to their probation advisor if they were interested in taking part. In total, 10 agreed to participate (90% male). All participants self-reported having committed criminal offences between the ages of 12–16 and participants did not receive any reward for their involvement in the study. Participants ranged in age from 22 to 53 years (*M* = 35.2, *S.D.* = 8.51). There was only one female participant within the sample. The lack of female representation within the sample is indicative of recidivism rates within England and Wales in that, approximately 84% of the reoffending population are male and therefore far less women than men are under the supervision of the probation service. Eight of the participants identified as White British whilst two participants self-identified as being Black British-Caribbean. Self-reported participant accounts suggest that the most common adolescent offences perpetrated among this participant group consisted of burglary, theft, robbery, and assault.

## 3 Materials

A semi-structured interview schedule was developed to elicit relevant information and guide the progression of the interviews. This schedule referred to seven core areas of exploration identified by the researchers as central to the issues of truancy and offending behaviour and identities. Participants were asked to reflect on their experience of truancy; parental knowledge of and attitudes towards absence from school; personal reflections on their juvenile criminal history; the impact that they felt their upbringing may have had upon their decision to commit crime; whether they favoured a particular type of criminal act (and if so why); how their offending behaviour had been affected by periods of incarceration; and finally, whether they were now able to identify the root cause of decisions to engage in of their juvenile offending behaviour.

### 3.1 Procedure

Ethical approval for the study was granted by the School Research Ethics Committee at the University of Huddersfield (UK). Participants were given a brief written description of the aims and objectives of this study contained within an information sheet. It was explained to them that participation would involve being interviewed and that their responses to questions asked would be audio recorded and later transcribed to allow for the purpose of analysis. It was also explained to participants that they should only provide information about criminal activities which they had personally engaged in between the ages of twelve and sixteen (the age at which young people in England and Wales are undertaking mandatory secondary education and schooling). They were informed that their responses would be subjected to subsequent analysis though would be anonymised at all times through use of a unique a participant number. An assurance was given that no disclosures regarding crimes for which they had not been investigated would be sought. All participants gave informed consent to take part in the study and gave their consent for interview responses to be included in research outputs on the basis that they were anonymised at all times. Each interview was conducted in accordance with the aforementioned interview schedule. Interviews ranged in length between 20 and 45 min (M interview length = 26.7 min). For all but one of the interviews, the only people in the room were the interviewer and the participant. The exception was the sole female in this study who agreed to be interviewed on the understanding that it took place in her home. Since the interviewer was male and the environment was not secure, she was interviewed in the presence of her social worker and probation officer who were in attendance prior to the researcher’s arrival and after his departure, but not in the same room during the interview to maintain her anonymity and encourage honesty in her responses. Specifically, the probation worker and social worker were sat with the primary researcher and the participant during the introduction (gaining informed consent) and the debrief, but where in a separate room during the interview. All ten interviews were audio-recorded and later transcribed. With a mean age of 35, it was believed that sufficient time had passed since the participants had experienced the cycle of truancy and youth offending such that they would therefore be better equipped to reflect upon their experiences with a view to identifying likely causes. The interviewees were asked to comment on the causes informing their decision to truant, their routes into and levels of involvement in crime, and motivation to pursue a deviant lifestyle. They were asked to account for their attraction to committing offences, about any drug and gang involvement, and their experiences and interactions with the criminal justice system. Finally, they were asked to reflect on their broader experiences to determine whether they could identify what they perceived to be the main cause(s) of their criminal behaviour, they were asked to speculate on why they made these choices and what they might have done differently on reflection. Participants were given the opportunity to review their transcripts and audio recordings to make any amendments. However, after doing so, no amendments or omissions were requested.

### 3.2 Analysis

Thematic analysis was conducted to identify and analyse emergent patters within the data (Braun and Clarke, 2006). Transcripts were examined in detail to identify, classify and interpret emergent themes. Each were later revisited to re-examine emerging themes which would later be explored as part of a shared experience offering insight into the environmental cues which may have motivated the participants to truant and engage in criminal activity during their adolescence/childhood. As part of this process of interpretation, each transcript was examined in the identical manner until commonality of themes were established and mapped across the entire data set.

## 4 Results

The results are presented in two parts, exploring the motivational themes identified for truancy and then criminal offending. Three themes of truancy motivation were identified: Boredom/disengagement, lack of punitive deterrence, and peer influence. For criminal behaviour, five themes of motivation were identified: Substance abuse, financial reward, developing a criminal identity, risk taking, and rejection and fear of authority.

### 4.1 Truancy

Truancy was identified as one of the most common themes among the ex-offenders, with all ten interviewees discussing times that they skipped school. Across the transcripts two main causes of truancy were frequently cited; boredom/disengagement and negative peer influence. Interestingly, none of the participants identified their parents as having any influence over their decisions to truant from school.

### 4.2 Boredom/disengagement

Boredom proved to be a powerful motivator for truanting, with eight out of ten participants identifying boredom as primary cause for truanting.

*“School’s crap! They weren’t teaching me what I wanted to know, so I couldn’t see the point.”* (Participant #09)

*“I’d get bored, so I’d … just get off and go and have a laugh with me mates, instead.”* (Participant #05)

*“No, I just couldn’t be bothered, innit? If you didn’t like a class that was on, or a teacher, or whatever, …. Wag school, innit, go and smoke a cigarette, or something”* (Participant #02)

Another contributory factor to the question of boredom was an inability to concentrate:

*“I’ve got a short attention span … I used to act up in class … I [got] bored very easily, causing mayhem … so they would throw me out.”* (Participant #03)

An additional reason suggested as having a negative impact on pupil engagement was rooted in the challenges faced by teachers who were trying to teach as well as poor perceptions of teaching quality.

*“I could say the teachers could have given me a bit more time, but in them days the classes was massive– they was struggling … you know what I mean?”* (Participant #06)

“… *the school that I was in– it was, like, I dunno– they’ve shut it down now, because the grades went down”* (Participant #04)

### 4.3 Lack of punitive deterrence

The failure to engage raised a significant challenge to discipline within the classroom which resulted in children skipping school because they realised they could truant with relative impunity. Four of those interviewed held the view that ineffective teachers and lack of authority were strong factors contributing to their truanting. The realisation that they could skip school with impunity served only to strengthen their tendency to engage in this behaviour.

*“Obviously, people did take the mickey in class and mess about … Teachers would [just] send you to the office, or kick you out of the class.”* (Participant #05)

### 4.4 Peer influence

From each of the interviews, it was clear that peer influence was a strong factor informing truancy, with all of the participants agreeing that peer pressure had played a part in motivating them into skipping school as part of the process of finding acceptance among their peers.

*“Following the crowd … it’s the norm(al) type of thing to do when you get to a certain age … everyone’s doing it … you just seem to follow.”* (Participant #01.)

*“Some people … do it to fit in … Someone could be brainy and actually like school, but because of [their] friends [who they think are] kind of cool …they … skip school just to fit in.”* (Participant #02)

*“I started hanging round with the naughty kids … it was like a form of trying to fit in.”* (Participant #07)

Another significant reason for truanting offered by several participants was the desire to belong to an older peer group. There was a definite attraction attached to mixing with older people who were no longer subject to the legal requirement of having to attend school. Many participants indicated that acceptance from older peers offered them excitement and the sense of being involved in something more adult.

*“ When I was twelve, I was knocking round with people of seventeen. They were not in school, they were off school, so I wanted to be with them … you’re looking up to them, aren’t you, you know what I mean?.. They’ve got a bit of money, you know? Or they’re selling drugs … you think: well …I like what they’re doing … Why am I sitting in class studying when I could be out [with them]?”* (Participant #03.)

*“When I was twelve, I was knocking round with people of seventeen. They were not in school, they were off school, so I wanted to be with them, and that was another reason I wouldn’t go to school.”* (Participant #03.)

*“When I got to … twelve, I was hanging round with sixteen-year-olds … smoking weed … all we seen around that area was people who sold drugs driving round [in] flash cars, not working, with loads of money, and it attracted me at that time … I thought, “I wanna be that person.”* (Participant #04)

### 4.5 Criminal behaviour

#### 4.5.1 Alcohol and substance misuse

Although not always being the direct cause for respondents’ criminality, substance misuse was present with all participant accounts of their formative years. Although nine of the ten participants admitted to using drugs, only one of the participants admitted to committing crime to fund their habitual use of drugs.

*“I smoked cannabis”.* (Participant #02)

*“You’re bouncing off each other …you’re trying to get (one over on the others) … and the next minute you take three ecstasy tablets and … you could be in hospital, you know what I mean? Like I’ve done it meself. I’ve had three ecstasy tablets all at once– from them ages, from twelve to sixteen, I was on ecstasy, coke … weed and alcohol, and that was every single weekend.”* (Participant #04.)

*“We’d go on the train, we’d get a big bottle of– three-litre bottle of coke, pour a litre out, put a litre of vodka in, mix it up and go on the train drinking it, then off. Do you know what I mean?...I actually smoked the first spliff at nine”* (Participant #04.)

*“… all I was wanting to do was go out with me friends and get drunk, stoned off weed, took– I took me first ecstasy tablet at twelve …”* (Participant #04.)

*“(From twelve to sixteen) I took cannabis, ecstasy, speed, cocaine. And then when I was 18, I started smoking crack and heroin.”* (Participant #07)

*“When I was fifteen, cannabis, sixteen, sixteen– and then I started ecstasy, then– oh, and LSD, as well– LSD when I was fifteen.”* (Participant #06)

This following interviewee also turned to prostitution at the age of 18 to fund her drug habit, she stated:

*“People that take drugs [commit] crime … to feed the drug habit … You have to do summat to get [the] stuff that you need to make yourself feel better. So, when you’re in that kind of a lifestyle [that’s when you] commit crime. I took drugs to change the way I felt.”* (Participant #07)

### 4.6 Financial reward

Financial reward was identified as a key motivator for committing crime by nine of the participants, even when the victims came from the same deprived background.

*“I’d rob addicts, I’d rob working girls, cos I was stood on the street and selling me body. So, I’d rob people that was vulnerable, who I knew I could rob, you know what I mean? Or I’d rob a drug dealer’s stash … I was always doing summat, whether it’d be going out and robbing someone, or shoplifting …But then, when I got [older] it sort of progressed … Then I was into robbing people in the street … I’d do anything, do you know what I mean? I’d do anything where I was going to get money … I didn’t have no limits. You know it comes to a point when anyone who commits crime … don’t have no limits, where they’ll just think … this is easy money. It’s easy money. And that– that’s the danger of it.”* (Participant #07.)

*“If you live in that kind of lifestyle and that’s where you’re growing up … you see …the guys in the fast cars … with the jewellery and that … you’re going to look at them [as] a role model, [rather than] your mum who’s working hard …and not got nothing to show for it ... [When you see your] mate’s been selling drugs for [a few] weeks, he’s got a new car, new jewellery … who you going to follow? (…) Who you going to follow?”* (Participant #02).

### 4.7 Developing a criminal identity

The development of a criminal identity was also considered by the participants and one theme which proved to be key was the achievement of status among criminal peers. One participant considered his status among his criminal peers to be more valuable than any financial reward.

*“Pretty much from twelve to sixteen I was quite dangerous with other kids …cos I’d hit people with poles, there’d be no stopping … I served me first sentence for hitting someone, me second one I bit their ear off. I was quite vicious … Back then, it was about me status as someone who was bad, so no one messed with (me) … so you could get what you want dead easy … I was quite passionate about what I wanted.”* (Participant #04)

Another factor inherent in the establishment of status was the replacement by some of their natural family with criminal peers. The involvement in crime created a sense of loyalty and belonging which was often perceived as missing from life at home.

*“At that age I just wanted to be with a gang, wanted to be with me mates, that was it. So, whatever they did, I did. [I belonged] with me friends [more than] me family.”* (Participant #03).

*“I started committing crime [because] I’d always seen crime in me life … like me mum selling drugs, or doing fraud. Money was always in our house. It was easy to come by, you didn’t really have to do nowt for it, so it was easy.”* (Participant #07)

### 4.8 Risk taking

Eight of the ten participants stated that a significant factor contributing to their juvenile offending was their enjoyment of taking risks as a form of reactional activity.

*“It was just that when you do something you feel a bit … better in yourself, bigger … more confident.”* (Participant #10)

*“I just liked that kind of lifestyle, found it attractive. It was fast, it was fun, it was chaotic … Normal people have got to go into work every day and that can be fucking boring, paying bills and all that– it all just looks shit. So, I wanted something that was exciting. I was … a thrill-chaser.”* (Participant #07)

*“I’ve done a lot of psychology reports from jail … and they had it down that I had sort of an ongoing personality disorder at that time. I had traits of it, because I was sensation-seeking. I used to go to Chester Bridge and climb up the top and jump off into the water, I used to go to Llangollen Straight (and be the) first person off …Major … risks, you know what I mean? We used to go …on the train … get a … three-litre bottle of coke, pour a litre out, put a litre of vodka in, mix it up and go on the train drinking it, then off (to Llangollen Straight) … (I was) fourteen ... There’s people died and everything in there, but at the time I was just, no, it doesn’t matter, I’m doing it. And it’s not just off the thing. If everyone’s off the thing I’m gonna take the next risk, you know what I mean?”* (Participant #04)

### 4.9 Rejection and fear of authority

A feature shared by all the participants was their utter disregard for being punished or imprisoned. Some participants admitted to not putting much thought into the consequences of their actions.

*“When you’re young, you don’t really think about it, do you?”* (Participant #01)

This was also evidenced when the participants were asked how they had coped when being jailed. Many participants asserted that incarceration was not a negative experience and did not consider it to be a strict punishment, consequently failing to deter future transgressions.

*“You’ll see people that you know, just like being back out in a way, (it’s) just like another big estate, jail … it’s no deterrent or nothing, it didn’t scare me.”* (Participant #01)

*“As soon as I went through the gates, I saw about eight guys I knew all playing football, they was telling me to try and get on this wing cos they was all on it, and that’s how it goes, so that first sentence (made me think) well, jail’s nothing! It’s easy, it’s like a little holiday.”* (Participant #02)

*“You’ll be expecting a hard time after what you see on the films and all that– and then you go there, it’s just totally different. You know what I mean, like? You’re kinda have a little laugh and enjoy yourself and you meet new friends– new criminals– so when you’re young and that, like, gaol don’t really bother you. Your sentences are low, your sentences are small, so you go in, come back out, like, and if you’re around that kind of, that lifestyle and those people that are in that lifestyle”* (Participant #02)

*“After like three weeks, I thought, ‘Is this it?’ You’re getting an education, you start getting your routine, I started feeling all comfortable, and then I started mixing with other criminals from other areas, like … Liverpool, Manchester … speaking to them about different crimes and learning things off them … I done eighteen months, I thought, ‘I’m a hardened criminal, now!’ … I thought, I could do that again and it led to me offending more.”* (Participant #04)

*“I thought, “Yeah, this is easy!”* (Participant #04)

“..But it was a factor in making me worse, because I knew that the outcome was not [worrying]– it couldn’t do nothing to me.” (Participant #04)

*“It did worry me when I was younger, yeah. You heard people saying it was this, it was that. It wasn’t how I thought it would be. It was completely different….. It weren’t too bad, to be honest.”* (Participant #10)

*“You’re like, “Oh, I’m out of here in four or six– what do they say? A shit, shower and a shave!”* (Participant #10)

However, some participant reported a different experience during incarceration, admitting to learning from the experience.

*“It was just a big shock…. It just changed me, everything– life, me outlook on life, me views about situations, about everything– it really has just changed me completely” .* (Participant #05)

*I was gonna use the time productively, get me life back on track, and walk out of the gates in four years' time with something to show for the time I’d lost….It helped me re-evaluate who I was... It’s given me an opportunity to better [myself]”* (Participant #08)

The court process, however, struck fear into most participants. They found the criminal justice system with its delays, procedures, pomp (Crown Court) and inexorable progress to be terrifying, daunting, intimidating and unpredictable.

*“If you go to trial you’ve got twelve strangers … coming up with the future of your life … You could be on that stand telling them the God’s honest truth and if them twelve people don’t believe you … then it’s ultimately them making a decision that could affect the rest of your life. And I don’t like the fact that you’re given ultimatums … that if you run a trial and … want to prove yourself …innocent, … [if you lose] you’re looking at a bigger sentence …and that shouldn’t be the case, cos you’ve got the trial to try to prove that you’re innocent. It’s [meant to be] innocent until proven guilty. But it’s actually the reverse of that. You’re guilty until you prove yourself to be innocent.”* (Participant #08)

*“Fuckin’ very scary, to be honest. It is, it’s really scary. It’s like your life is in someone else’s hands.”* (Participant #7).

## 5 Discussion

The primary aim of this study was to retrospectively explore factors which motivated adolescents’ involved in antisocial and criminal behaviour to truant from school and engage in criminality through qualitative interviews with a sample of recently released adult prisoners. Thematic analysis uncovered several key themes related to substance misuse and broader enjoyment of risk-taking behaviours, financial gain and development of a criminal status, alongside fear and rejection of authority. Most participants cited boredom as an important determinant of their decisions to truant from school. Interestingly, despite many participants describing coming from disrupted family backgrounds, none cited parental or familial neglect as being a key causal influence for their truanting behaviour. As such, the present findings obtained through retrospective interviews with recently released adult offender, contradict prior research that found more attentive parenting styles can discourage children from truanting (McNeal, Jr, 1999; Okorodudu, 2010). It is of course entirely possible that participants in our study chose not to externalise their behaviour and attributed their actions to more overt experiences recalled. Most participants reported drug taking behaviours including specifically, smoking cannabis. Research into substance misuse has demonstrated not only that juvenile indulgence begins at an early age but that gateway drugs like cannabis can lead to more sever substance misuse and addiction to substances, such as heroin, in later life (Lynskey, 2003). This was evident in an account given by the only female participant in the study, who stated that she broadened her experimentation with drugs at the age of eighteen by moving onto crack-cocaine and heroin; and ultimately turned to prostitution to fund her subsequent addiction. However, other researchers are less certain of such a link. Nkansah-Amankra and Minelli (2016) argue that although early drug use appeared to increase the risk of more severe drug use in later life, in many cases adolescent drug experimentation decreased the prevalence of substance misuse in adulthood. According to Moffitt (1993), there are two types of antisocial behaviours which lead to delinquency: adolescence-limited antisocial behaviour and life-persistent antisocial behaviour. The first usually peaks at about the age of seventeen and is therefore temporary and situational. The second is persistent and stable; and it is this type which appears to have afflicted the participants in this study since they were still offending into their twenties and thirties (and in one case into his fifties). Most of the participants had gained lengthy criminal records as adults, with offences ranging from selling Class A drugs to extreme acts of violence. As Corr (2014) pointed out, there is a danger of viewing juveniles solely in relation to their offending histories without considering it bilaterally with their often deprived and complex social histories. Most participants in the current study reported committing criminal acts while truanting, with several reporting peer-respect to be their primary motivation. These findings align with researchers who report vast evidence of the importance of bonds formed with deviant and offending peers in the commission of crime and development of more sustained criminal identities (Boduszek et al., 2013, 2016, 2021; Sherretts and Willmott, 2016; Spink and Woodfield, 2019). Similarly, the importance of in-group ties and affection held towards criminally active peers is shown to be an important determinant of criminal identity among offending (Spink et al., 2020). According to Manski (1993) there is an intrinsic (or endogenous) drive which gives rise to bidirectional influences. Gaviria and Raphael (2001) add further weight to support this argument by illustrating how significant social interaction is to the question of delinquency and offending. In the current study almost all participants reported engaging in crime whilst truanting, identifying social mimicry as an important feature in their offending and deviant behaviour.

Alongside the participant that explained her reason for committing crime was the acquisition of wealth to fund her drug use, almost all of the other participants referred to the pursuit of financial gain as a key motivator of their offending, with a number of those interviewed explaining that they were attracted to committing crime after observing older boys who appeared to be profiting from selling drugs and committing crime. The assertion that many juveniles offend in pursuit of financial reward is supported by vast previous theorising and empirical research. Levitt (1998) and Mocan (2005) argue that the economic model of crime is not restricted to adult offenders and often acts as a gateway into criminality, especially among disadvantaged youth.

Almost all participants agreed that taking risks had been an important feature in their offending while truanting. The current findings therefore align with a wealth of previous studies that found youth offending is frequently motivated by thrill seeking and the desire to alleviate boredom through a sense of risk-taking adventure (Boyer and Byrnes, 2009; Pfefferbaum and Wood, 1994; Reniers et al., 2016). Studies with adolescent rioters in England, and separately in Northern Ireland, both obtained evidence that a range of offences including criminal damage, theft, and riotous disorder were described by rioters as rooted in the desire for risky reactional activity (Leonard, 2010; Willmott and Ioannou, 2017). Research exploring causal underpinnings for adolescent fire-setting behaviour also concluded risk-taking recreation was a key motivation for engaging in this behaviour (Mojtahedi et al., 2017). It appeared that some participants were motivated to offend in order to establish a reputation or criminal status as a way of disguising vulnerability and feeling of low self-esteem. Some participants expressed that when raised in a socially deprived area, the only way to survive was by achieving a heightened criminal status. A minority of participants stated that use of violence towards other adolescents was a conscious act that was underpinned by a lot of thought and effort which the express intention of building and assuming a criminal identity. Similarly, the experiences endured by the only female participant in the study drove her to consciously mask her perceived social inadequacies through fear of isolation and rejection from being the only black child in her school. Much research accords with these assertions that explain individual motivations for engaging in crime, especially among adolescence actively involved in the process of forming and shaping their identity. It is empirically well established that the development of criminal identity is seeded by a lack of social acceptance, environmental deficits, familial/peer disavowal, low self-esteem and the search for acceptance through criminal association (Boduszek et al., 2013, 2016; Sherretts and Willmott, 2022; Spink et al., 2020). Regardless of the reasons offered by the participants in this study for committing crime, it becomes clear that punishment engendered little fear. This presents a challenge for state authorities, educators and criminal justice authorities to overcome in that the multiplicity of factors which merge to develop an offender and offending identity, are not only diverse but also complex and vary in significance between individuals. The mechanisms and efficacy by which identity can be changed once constructed is also highly variable (see Barnett et al., 2021).

Reflective discussions with former adolescent offenders provided an ecologically reliable understanding about the fundamental causes of juvenile offending and truancy, from which practical implications can be drawn. Moreover, the findings highlight the need for greater investment in educational and recreational provisions within areas considered to be ‘economically impoverished’ (and also correctional facilities, see Turner et al., 2021) as a potential crime prevention strategy—given the interviewees' reports of poor educational experience, boredom, and the attraction to financial incentives as primary motivations for truancy and criminality. However, the study ought to be considered in light of a number of limitations. For example, it was conducted using a small group of predominately adult male participants recently released from prison. This means that any findings should be viewed as directions towards future research and not as an end it itself. The study also spotlights retrospective adolescent experiences and motivations among a sample of participants with complex childhood and adult lives which may be difficult for them to disentangle. Future research should therefore attempt to explore retrospective motivations for truancy and adolescent criminality among a cohort of adults who did not engage in adult criminality and have not experienced incarceration during adulthood, in an effort to understand how they were able to desist from such juvenile criminal activities, identities and lifestyles. Secondly, the use of a semi-structured interview schedule reduced opportunities for the researchers to sufficiently stray away from the predefined questions set out for each participant based upon predefined study aims and parameters. This restriction was particularly evident when participants started to open up and disclose information which did not relate to the fixed age parameters of 12–16 years. Finally, the researchers also acknowledge the possibility that what the participants chose to disclose may not have been entirely truthful about the full extent of their criminal histories or childhood experiences.

## 6 Conclusion

Psychological research is routinely providing practical knowledge around the management and prevention of crime (e.g., Wainwright and Mojtahedi, 2020: Willmott et al., 2021; Mojtahedi et al., 2018, 2019) and the present study follows suit. Despite the adoption of an alternative study design and method by which factors contributing adolescent truancy and offending can be assessed, the study findings obtained here support conclusions drawn in a wealth of past research surrounding factors which contribute to such juvenile behaviours. That said, the methodological approach undertaken affords offenders a voice in explaining their own deviance and offending in a way that other research has not. Traditional methods have historically failed to answer the many questions juvenile offending have posed and consequently had negligible impact on the nature of offending or efforts to reduce recidivism. Taken together, the findings of the current research and those obtained in a wealth of previous studies suggest no single agency is equipped to offer the multitude of interventions required to overcome the causes underlying truancy and offending. In fact a multi-agency approach whereby educators, criminal justice practitioners and various other state authorities work together to address the causal influences outlined above, is likely to offer the greatest chance of diverting young people away from engaging in adolescent delinquency, and subsequent criminal lifestyles in adulthood. The only way that this can be achieved is by assembling relevant and influential agencies which are properly equipped with the apparatus needed to nurture a positive outcome. It is therefore this study’s contention that the only way to deal with the co-dependent issues of truancy and juvenile offending is to increase investment in multi-agency involvement, which is properly regulated, mutually supportive, and appropriately organised.

## Declarations

### Author contribution

Stephen Filkin: Conceived and designed the experiments; Performed the experiments; Analyzed and interpreted the data; Contributed reagents, materials, analysis tools or data; Wrote the paper.

Dara Mojtahedi: Conceived and designed the experiments; Analyzed and interpreted the data; Contributed reagents, materials, analysis tools or data; Wrote the paper.

Dominic Willmott: Conceived and designed the experiments; Analyzed and interpreted the data; Wrote the paper.

### Funding statement

This research did not receive any specific grant from funding agencies in the public, commercial, or not-for-profit sectors.

### Data availability statement

The authors do not have permission to share data.

### Declaration of interest’s statement

The authors declare the following conflict of interests: The corresponding author of this manuscript (Dr Dominic Willmott) is on the editorial advisory board of Heliyon: Psychology Section.

### Additional information

No additional information is available for this paper.
